# Role of interleukin 4 and its receptor in clinical presentation of chronic extrinsic allergic alveolitis: a pilot study

**DOI:** 10.1186/2049-6958-8-35

**Published:** 2013-05-30

**Authors:** Martina Sterclova, Radoslav Matej, Petra Mandakova, Jelena Skibova, Martina Vasakova

**Affiliations:** 1 Department of Respiratory Diseases, ThomayerHospital, Videnska 800, Prague 4 140 00, Czech Republic; 2 Department of Pathology and Molecular Medicine, ThomayerHospital, Videnska 800, Prague 4 140 00, Czech Republic; 3 Department of Pathology and Molecular Medicine, 2nd Medical Faculty of Charles University, University Hospital Motol, V Úvalu 84, Prague 5 150 06, Czech Republic; 4 Department of Medical Statistics, Institute of Clinical and Experimental Medicine, Videnska 1958/9, Prague 4 140 21, Czech Republic

**Keywords:** Chronic extrinsic allergic alveolitis, High resolution computed tomography score, Interleukin 4, Interleukin 4 receptor, Sarcoidosis

## Abstract

**Background:**

Th-2 cytokine milieu including interleukin 4 (IL-4) was detected in fibrotic lung diseases. Chronic extrinsic allergic alveolitis (EAA) may be also accompanied by marked fibrogenesis. The aim of this study was to determine if IL-4 and its receptor (IL-4R-alpha) play any role in the clinical presentation and pathogenesis of chronic EAA.

**Methods:**

Twenty patients originally investigated for interstitial lung disease and finally diagnosed affected with chronic EAA and sarcoidosis were prospectively enrolled into the study. Concentrations of IL-4, IL-4R-aplha and total protein were assessed in the bronchoalveolar lavage fluid (BALF) of all enrolled subjects as well as high resolution computed tomography (HRCT) scores and pulmonary function tests.

**Results:**

BALF IL-4R-alpha and total protein concentrations were significantly higher in chronic EAA patients (p < 0.05). Concentrations of BALF IL-4R-alpha were significantly higher in men than in women (p < 0.05) in EAA group. Total protein BALF levels were significantly elevated in ex-smokers with EAA compared to nonsmokers (p < 0.05). A positive correlation (p < 0.01) between IL-4R-alpha BALF concentrations and HRCT interstitial scores were observed in chronic EAA group; the IL-4R-alpha/total protein ratio showed the same significant positive correlation. A negative correlation between lung function results and IL-4R-alpha, and IL-4R-alpha/total protein as well, was also found (p < 0.05).

**Conclusions:**

We suggest a clinical relevance for the IL-4/IL-4R axis in the etiopathogenesis of chronic EAA. IL-4R-alpha could serve as a potential biomarker of lung fibrogenesis.

## Background

Even though extrinsic allergic alveolitis (EAA) is among the most frequently diagnosed interstitial lung diseases, pathogenetic events leading to lung involvement need to be elucidated. Originally, immune complex deposition and related injuries were thought to play a major role in EAA development [1]. Further studies showed the potential of adoptive transfer of EAA by Th1 lymphocytes**,** and the influence of mainly Th1 cytokines and chemokines on disease manifestation has been reported (namely: interferon gamma, and interferon gamma induced protein 10) [2]. Later, Th2 cytokine milieu was found to play a role in the etiopathogenesis of pulmonary fibrosis in chronic EAA patients [3]. Interleukin-4 (IL-4) is a Th2 cytokine and has been found to take part in the pathogenesis of fibrosis in the ‘bleomycin model,’ as well as in idiopathic pulmonary fibrosis (IPF) patients. IL-4 is thought to be an important chemotactic factor for fibroblasts and it can enhance both their proliferation and collagen production. Membrane bound and soluble IL-4 receptors (IL-4R) have been described, especially IL-4R-alpha. The soluble form of IL-4R can have both antagonist activity or can act as a carrier of IL-4 and its biological properties [4]. Furthermore, IL-4 was found to act as a proangiogenic factor in hypoxic conditions [5]. Despite pulmonary fibrosis is the main histopathological and radiological feature of chronic EAA, the role of IL-4 and IL-4R in EAA pathogenesis has not been studied yet.

The aim of this study was to determine if IL-4 and IL-4R play any role in chronic EAA pathogenesis by: (i) comparing bronchoalveolar lavage fluid (BALF) concentrations in chronic EAA and sarcoidosis patients (sarcoidosis being considered a typical Th1 mediated disease) and (ii) looking for correlations among IL-4 and IL-4R BALF concentrations and clinical parameters of patients with chronic EAA (gender, smoking status, pulmonary function tests, BALF differential cell counts, high resolution computed tomography pattern of the disease).

## Methods

### Patients

Fourteen patients (7 men / 7 women, aged 59 ± 11 years, 7 non-smokers and 7 ex-smokers) originally investigated for interstitial lung disease and finally diagnosed with chronic EAA were prospectively enrolled into the present study. A second control group was formed by six patients diagnosed with sarcoidosis according to the European Respiratory Society/American Thoracic Society/ World Association of Sarcoidosis and Other Granulomatous Disorders (ERS/ATS/WASOG) criteria (4 men / 2 women, aged 46 ± 13 years, 2 non-smokers, 3 ex-smokers, 1 current smoker, 4 patients sarcoidosis stage 2, 2 patients stage 4) [6]. All participants gave informed consent and the study was approved by Ethics Committee of the ThomayerHospital and Institute of Clinical and Experimental Medicine, Prague, Czech Republic. As part of the diagnostic work-up, all patients provided a detailed medical history, underwent blood tests including exclusion of laboratory signs of autoimmune diseases, pulmonary function tests (spirometry, lung diffusing capacity for carbon monoxide-Dl_co_), bronchoscopy with bronchoalveolar lavage and transbronchial biopsy, a high resolution computed tomography of the chest (HRCT) and a surgical lung biopsy, when diagnosis remained uncertain even after the above mentioned diagnostic procedures (8 EAA patients, 4 sarcoidosis patients). Since there are no universally accepted diagnostic criteria for EAA, the criteria used in this study are summarized in Table 1. Only patients with probable/definite chronic EAA were included in the study group.

**Table 1:** Diagnostic criteria for chronic EAA used at author’s institution

History of exposure to inhalation antigen	+/−
Crackles	+
DL_co_	↓
BALF lymphocytosis	+/−
BALF CD4/CD8	↓/normal/↑
HRCT pattern	Centrilobular nodules
	GGO
	Mosaic perfusion
	Condensations
	Interstitial septa thickening
	Honeycombing
Histology pattern	Granuloma, OP, DIP, NSIP, UIP

*BALF* bronchoalveolar lavage fluid, *DIP* desquamative interstitial pneumonia, *DL*_*co*_ lung diffusing capacity for carbon monoxide, *GGO* ground glass opacities, *HRCT* high resolution computed tomography, *NSIP* nonspecific interstitial pneumonia, *OP* organising pneumonia, *UIP* usual interstitial pneumonia.

**Possible chronic EAA**: negative history of exposure to inhalation antigen+ BALF lymphocytosis+typical HRCT pattern/histology pattern (see above in the Table).

**Probable chronic EAA**: positive history of exposure to inhalation antigen+typical HRCT/histology pattern (see above in the Table).

**Definite chronic EAA**: positive history of exposure to inhalation antigen+BALF lymhocytosis+typical HRCT/histology pattern (see above in the Table).

### Bronchoalveolar lavage

Bronchoalveolar lavage (BAL) was performed during the fiberoptic bronchoscopy under local anesthesia. The bronchoscope was wedged into the segmental part of the middle lobe. Five fractions of 50 ml of lukewarm saline were instilled and after each instillation gently aspirated. Only samples with recovery > 50% were used (mean recovery per one fraction 57 ± 20%). The retrieved fluid was mixed in a sterile container before division for further investigation. To assess the BAL fluid (BALF) cell differential counts, 5 ml of BALF were centrifuged and standard cytological sample was prepared with Giemsa-Romanovski staining. Cells were counted using a light microscope and a 125× objective; cells in 100 fields were counted and percentages of alveolar macrophages, lymphocytes, neutrophils and eosinophils were estimated.

Concentrations of IL-4R-alpha (CD-124) in the bronchoalveolar lavage were determined using the ELISA method (Uscn Life Science Inc, Houston, USA) according to manufacturer’s instructions. A FlowCytomix human Th1/Th2 11plex Ready-to-Use Kit (Bender MedSystems Vienna, Austria) was used for quantitative detection of IL-4 in the supernatants obtained from the BALF according to the manufacturer’s instructions.

The concentration of total protein was measured spectrophotometrically using a bicinchoninic acid assay (BCA kit; Pierce, Rockford, USA) following the manufacturer’s instructions. To reduce the impact of using different BALF dilutions, the IL-4/total protein and IL-4R/total protein ratios were used to validate correlations among clinical parameters and interleukin’s BALF concentrations.

### Pulmonary function tests

Forced vital capacity (FVC) and forced expiratory volume in one second (FEV_1_) were obtained during initial investigations and at the one year follow up using a ZAN 100 Flowhandy II (Inspire, Oberthulba, Germany). ERS/ATS performance criteria were used for the test [7]. The predicted spirometry values complied with those of the European Coal and Steel Community [8]. Diffusing capacity for CO (Dl_co_) was obtained using a ZAN 300 CO-diffusion (Inspire, Oberthulba, Germany) on the same day as spirometry was performed. The Dl_co_ was measured using the single-breath method. Values were expressed as a percentage of the predicted value.

### High resolution computed tomography

All enrolled subjects underwent a HRCT of the chest during the initial investigation. HRCT scans were performed using a LightSpeed VCT XT scanner. HRCT alveolar and interstitial scores were assessed by a pulmonologist experienced in radiology and carried out according to Gay SE et al. (Table 2) [9].

**Table 2:** HRCT scoring system according to Gay SE et al.*

**Score**	**Alveolar**	**Interstitial**
0	No alveolar disease	No interstitial disease
1	GGO < 5%	Septal thickening, no honeycombing
2	GGO < 25%	HC/ septal thickening < 25%
3	GGO 25-49%	HC/septal thickening 25-49%
4	GGO 50-75%	HC/septal thickening 50-75%
5	GGO > 75%	HC/septal thickening > 75%

Scored fields = peak of aortic arch, tracheal bifurcation, most prominent convexity of the right ventricle, and peak of the right cupola of diaphragm. Mean interstitial and alveolar scores were counted.

*GGO* ground glass opacity, *HC* honeycombing.

*[9]**.**

### Statistical methods

Results are expressed as the mean ± standard deviation (SD)**/** median [min-max value]. Differences between two variables were assessed using the Mann–Whitney U test. The Spearman’s correlation was used to analyze correlations among variables. For all statistical methods, p < 0.05 was regarded as significant.

## Results

Results of pulmonary function tests and HRCT alveolar and interstitial scores in both groups (chronic EAA vs. sarcoidosis) are presented in Table 3.

**Table 3:** Results of pulmonary function tests, HRCT alveolar and interstitial score of chronic EAA and sarcoidosis patients

	**Chronic EAA**	**Sarcoidosis**	**p**
FVC (% e.v.)	79,8 ± 17,8	86,2 ± 22,4	> 0,05
	79 [55–121]	92,5 [42–101]	
FEV_1_ (% e.v.)	84,0 ± 18,2	81,2 ± 19,9	> 0,05
	81,5 [53–124]	87,5 [42–98]	
**Dl**_**co**_**(% e.v.)**	**47,9 ± 19,7**	**62,8 ± 17,5**	**< 0,05**
	**42 [30–91]**	**55 [46–98]**	
HRCTa	1,7 ± 1,0	2 ± 1,6	> 0,05
	1,5 [0–3]	2 [0–4]	
HRCTi	1,9 ± 1,0	1,5 ± 1,3	> 0,05
	2 [1-4]	1 [0–3]	

*Dl*_*co*_ lung diffusing capacity for carbon monoxide, *e.v.* expected value, *FEV*_*1*_ forced expired volume in 1 second, *FVC* forced vital capacity, *HRCTa* high resolution computed tomography alveolar score, *HRCTi* high resolution computed tomography interstitial score. Values are presented as mean ± SD/ median [min-max value].

BALF differential cell counts, total protein concentrations, IL-4 and IL-4R-alpha concentrations are summarized in Table 4. Correlations among BALF total protein, IL-4 and IL-4R-alpha concentrations and clinical parameters (gender, smoking status, pulmonary function tests, HRCT, BALF differential cell counts) are summarized below.

**Table 4:** BALF cell counts, total protein concentrations, IL-4 and IL-4Ralpha concentrations in chronic EAA and sarcoidosis patients

	**Chronic EAA**	**Sarcoidosis**	**p**
Alveolar macrophages (%)	65,1 ± 24,8	81,0 ± 9,9	> 0,05
	72,5 [12–94]	80,5 [72–91]	
Polymorphonuclear cells (%)	6,4 ± 6,6	4,5 ± 5,1	> 0,05
	5 [1-28]	2,5 [1-12]	
Lymphocytes (%)	19,5 ± 21,9	15,8 ± 8,4	> 0,05
	8 [2–74]	15,5 [8/24]	
Eosinophils (%)	7,3 ± 8,1	1,3 ± 1,1	> 0,05
	6,5 [0–25]	0,5 [0–4]	
IL-4 (pg/ml)	34,9 ± 31,0	25,5 ± 18,0	> 0,05
	29,2 [0–96,4]	25,8 [0–50,5]	
**IL-4Ralpha (pg/ml)**	**1189,7 ± 970,5**	**302,7 ± 212,1**	**< 0,05**
	**1039,5 [33–2365]**	**321,5 [49–589]**	
**Total protein (μg/ml)**	**221,1 ± 214,7**	**80,6 ± 37,2**	**< 0,05**
	**138,9 [39,7-767,5]**	**72,7 [43,2-138,4]**	

*IL-4* interleukin 4, *IL-4Ralpha* alpha subunit of interleukin 4 receptor.

Values are presented as mean ± SD/ median [min-max value].

**Figure 1:**
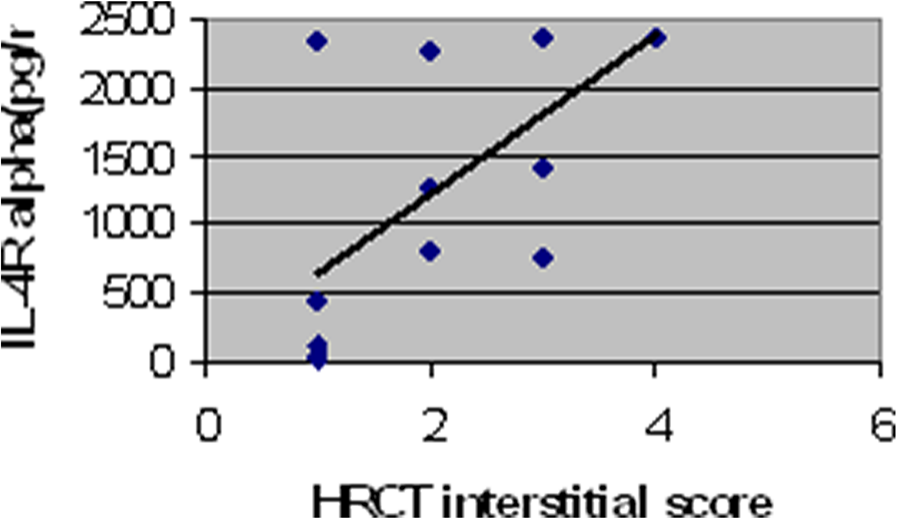
**Positive correlation between HRCT interstitial score and IL4R BALF concentrations in chronic EAA patients.** BALF,bronchoalveolar lavage fluid; EAA, extrinsic allergic alveolitis; HRCT, high resolution computed tomography; IL-4R, interleukin 4 receptor (pg/ml).

**Figure 2:**
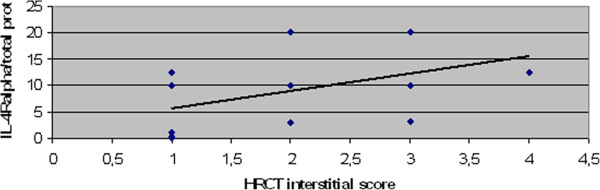
**Positive correlation between HRCT interstitial score and IL-4R/total protein in BALF of chronic EAA patients.** BALF,bronchoalveolar lavage fluid; EAA, extrinsic allergic alveolitis; HRCT, high resolution computed tomography; IL-4R, interleukin 4 receptor (pg/ml).

**Figure 3:**
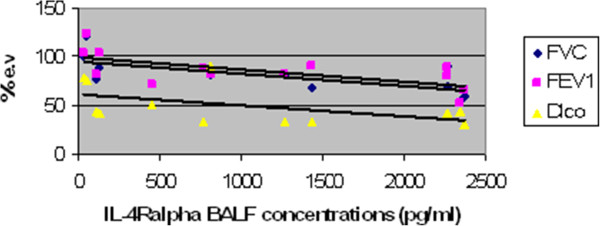
**Negative correlations among IL-4R concentrations in BALF and FVC, FEV**_**1 **_**and DL**_**co **_**in chronic EAA patients.** BALF, bronchoalveolar lavage fluid; DL_co_, lung diffusing capacity for carbon monoxide, EAA, extrinsic allergic alveolitis; e.v., expected values; FEV_1_, forced expired volume in 1 second; FVC, forced vital capacity; HRCT, high resolution computed tomography; IL-4R, interleukin 4 receptor (pg/ml). FVC, FEV_1_, DL_co_ expressed in % of expected value.

**Figure 4:**
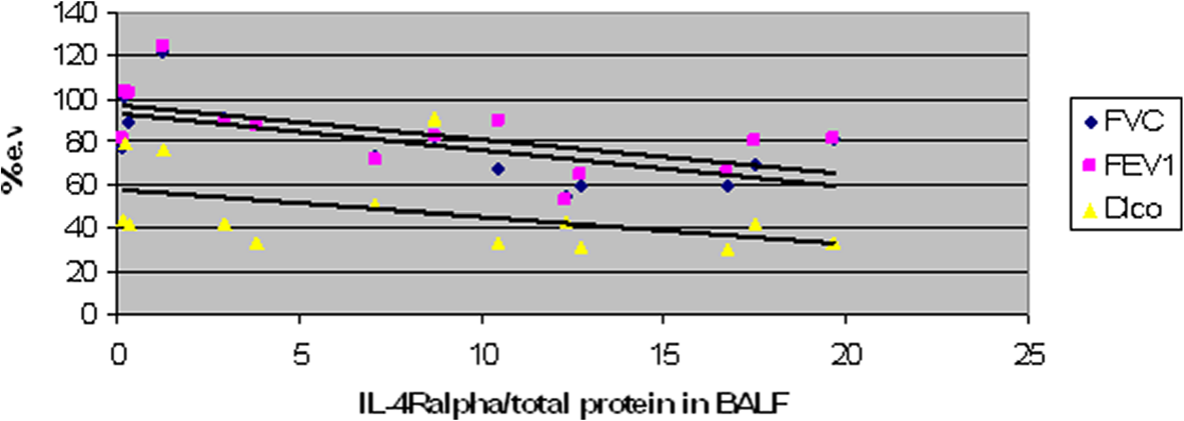
**Negative correlations among IL-4/total protein in BALF and FVC, FEV**_**1 **_**and DL**_**co **_**in chronic EAA patients.** BALF, bronchoalveolar lavage fluid; DL_co_ lung diffusing capacity for carbon monoxide; EAA, extrinsic allergic alveolitis; e.v., expected values; FEV_1_, forced expired volume in 1 second; FVC, forced vital capacity; HRCT, high resolution computed tomography; IL-4R, interleukin 4 receptor (pg/ml). FVC, FEV_1_, DL_co_ expressed in % of expected value.

Concentrations of BALF IL-4R-alpha were significantly higher in men diagnosed as EAA than in women (p < 0.05). BALF total protein levels were significantly elevated in ex-smokers with EAA compared to non-smokers (p < 0.05). Positive correlation (p < 0.01) between IL-4R-alpha BALF concentrations and HRCT interstitial scores was observed in the chronic EAA group (R=0,74). The IL-4R-alpha/total protein ratio also showed the same significant positive correlation (R=0,71) [Figures 1 and 2]. Negative correlations among FVC/IL-4 Ralpha(R= −0,73), IL-4 Ralpha/total protein (R=−0,66), FEV_1_/IL-4 R alpha (R= −0,70) , IL-4 R alpha/ total protein (R= −0,63), Dl_co_ (IL-4 R alpha :R=−0,67, IL-4R alpha/total protein: R= −0,57) and IL-4R-alpha as well as IL-4R-alpha/total protein were found (p < 0.05) [Figures 3 and 4]. No further significant correlations were observed in the chronic EAA group.

As to sarcoidosis patients, correlations were not assessed because of the small number of enrolled patients.

## Discussion

The present study was aimed to determine whether Th2 cytokine IL-4 and its soluble receptor IL-4R-alpha take part in the fibrogenic process seen in chronic EAA, which is thought to exhibit a Th2 cytokine milieu. Data obtained from chronic EAA patients were compared to sarcoidosis patients. Sarcoidosis is considered to be a typical Th1 disease, moreover studies of IL-4R gene polymorphisms have showed no association with sarcoidosis, and no differences were found in IL-4 BALF concentrations from sarcoidosis patients compared to healthy controls [10,11].

In this study the patients groups differed in age, and this is not surprising as the usual age of sarcoidosis manifestation is < 40 years. None of chronic EAA patients were considered professional EAA, and it could be related to lower age [12].

Initially, BALF concentrations of IL-4 and IL-4R-alpha were compared in chronic EAA and sarcoidosis patients. Even though the difference was not statistically significant, higher IL-4 concentrations were observed in EAA group. Previous studies have documented the role of IL-4 as well as interferon gamma in the pathogenesis of EAA, both cytokines being produced by non-immune cells [13]. The effect of IL-4 on fibrosis development in EAA patients varies form study to study. Some authors even documented a remarkable improvement in murine EAA after IL-4 administration, while others did not find such effect for this cytokine [14,15]. Even though IL-4 augments the effects of fibroblasts in *in vitro* studies, its contribution to fibro-proliferation in EAA patients has not been studied. Nevertheless studies performed in IPF patients have shown a clear role for IL-4 in fibrogenesis; distinct polymorphisms of the gene for IL-4 have been associated with more severe radiological involvement [16,17]. Because skewing of the immune response from Th1 in the reversible stages of the disease to Th2 in the later fibrotic stages has been observed, we might think that the role of IL-4/IL-4R could fluctuate according to the manifestation of the disease. Chronic EAA may share radiologic as well as histological features of usual interstitial pneumonia and perhaps IL-4 can play a similar role in IPF and chronic EAA. According to the studies by Pechkovsky et al., IL-4 is able to induce further expression of IL-4R, which together with the above mentioned findings may suggest a link to higher concentrations of IL-4, resulting in higher concentrations of IL-4R-alpha and more pronounced fibro-proliferation [18].

More severe impairment of Dl_co_ (p < 0.05), significantly higher BALF total protein concentrations and IL-4R-alpha (p < 0.05) were accompanied by non-significantly higher HRCT interstitial scores in chronic EAA patients. We can speculate that BALF soluble IL-4R-alpha concentrations perhaps could be an even more sensitive marker of fibrosis than the more commonly mentioned HRCT [19,20].

The second aim of this study was to determine if an association between IL-4/IL-4R-alpha and EAA etiopathogenesis could be supported by a correlation between BALF concentrations and the clinical presentation of the disease (i.e., BALF differential cell count, pulmonary function tests and HRCT scores). Because there were only six patients in the sarcoidosis group, correlations were not performed for this group. A positive correlation between HRCT interstitial scores and IL-4R-alpha BALF concentrations was observed, as well as a negative correlation relative to FVC, FEV_1,_Dl_co_ and IL-4R-alpha BALF concentrations. The relevance of this finding was supported by the same results of IL-4R-alpha adjusted for BALF total protein concentrations. These observations might suggest that IL-4R-alpha acts like a carrier of biological functions of IL-4 in chronic EAA with HRCT signs of fibrotic lung impairment.

BALF total protein concentration in EAA patients with a history of smoking was significantly higher than in non-smoking patients. This findings agree with the results of Sato et al., even though their study measured albumin concentrations [21]. Others have found lower concentrations of proteins and mRNAs in smoking subjects [22,23]. This discrepancy may be due to the fact that, while acute exposure to cigarette smoke reduces blood-airway permeability, chronic exposure to smoke may disrupt the endothelial barrier integrity and thus influence endothelial permeability [24,25]. Thus a history of smoking could be an important factor influencing concentrations of various proteins in BALF.

It can be objected that smoking (or exposure to nicotine) was thought to be a protective factor for granulomatous involvement in EAA patients [26]. However, Furuiye et al. showed that long term smoking may lead to more pronounced fibrogenesis even in EAA subjects, probably because cigarette smoke is composed by many substances, some of which could possibly have pro-fibrotic effects. It also should be kept in mind that smoking is a risk factor for other interstitial lung diseases including IPF and rheumatoid arthritis associated interstitial lung disease [27].

In our study group BALF IL-4R-alpha concentrations were found to be higher in men with EAA than in women. There is no consistent information concerning gender effect on EAA manifestation, perhaps because most studies found in literature concern special types of EAA (farmer’s lung, pigeon breeder’s lung, etc.). On the other hand, interstitial lung diseases, like IPF, dominate in the male population. Of course, smoking status has to be considered, and smoking is still more common in men than in women in most countries including the Czech Republic. However, the effect of gender on immune reactions has been reported by many authors. Pinzan et al. observed different immunological reactions in men and women infected by Paracoccidioidesbrasiliensis infection [28]. According to results of this study, men who were more often infected presented with a dominant Th2 immune response, while women exhibited a Th1 immune response. Different immune responses are usually explained by the influence of estrogen or testosterone on different T cell populations, resulting in different cytokine milieu [29,30]. Murine studies have shown that not only gender of infected mice, but also IL-4 alpha status can influence outcomes of some infectious diseases [31]. As mentioned above, higher BALF concentrations were associated with more severe radiological and pulmonary function test deterioration. It is possible that differences in IL-4R-alpha expression between males and females are part of different manifestations of the condition and perhaps outcomes of some interstitial lung diseases.

In our opinion the present study has several limitations. First of all, the number of enrolled patients was small, however the study was performed prospectively and consecutive patients suspected of interstitial lung disease were enrolled, among which we finally chose those with a definite diagnosis of chronic EAA or sarcoidosis. We assessed the concentrations of IL-4R-alpha, which is not only part of IL-4R, but also of the IL-13 receptor. Nevertheless, the soluble form of IL-13R was formed by IL-13R-alpha-2 and IL-4R-alpha may be part of a membrane bound IL-13R.

## Conclusions

BALF IL-4R-alpha concentrations were significantly higher in chronic EAA than in sarcoidosis patients. Furthermore, their BALF IL-4R-alpha concentrations correlated with the extent of fibrotic changes assessed using HRCT and with pulmonary function tests impairment, suggesting a relevance to the pathogenesis of chronic EAA. The use of IL-4RA as a potential fibrogenic marker in chronic EAA patients should be further assessed. Its elevated concentrations found in BALF obtained at the time of diagnosis could select patients with more fibrotic potential. Such a finding can be useful in treatment decision and in selected cases could lead to earlier discussion about possible need for lung transplantation. Higher total protein BALF concentrations in ex-smokers may suggest higher endothelial cell permeability, which may affect concentrations of other proteins in BALF as well. BALF concentrations of IL-4R-alpha were significantly higher in males with chronic EAA. The gender effect on chronic EAA manifestation needs further study.

## Competing interest

Authors declare no competing interests.

## Authors’ contributions

MS designed the study, contributed to data acquisition, performed HRCT scoring and drafted the manuscript. RM and PM contributed to BALF investigation. JS made statistical analysis of the data. MV performed bronchoalveolar lavages, revised the manuscript and contributed to the draft of the manuscript. All authors read and approved the final manuscript.
